# Versatile Oxidase and Dehydrogenase Activities of Bacterial Pyranose 2-Oxidase Facilitate Redox Cycling with Manganese Peroxidase *In Vitro*

**DOI:** 10.1128/AEM.00390-19

**Published:** 2019-06-17

**Authors:** Peter L. Herzog, Leander Sützl, Beate Eisenhut, Daniel Maresch, Dietmar Haltrich, Christian Obinger, Clemens K. Peterbauer

**Affiliations:** a Department of Food Science and Technology, BOKU—University of Natural Resources and Life Sciences, Vienna, Austria; b Department of Chemistry, BOKU—University of Natural Resources and Life Sciences, Vienna, Austria; University of Toronto

**Keywords:** actinobacteria, lignin degradation, manganese peroxidase, phylogeny, pyranose oxidase

## Abstract

Pyranose 2-oxidase (POx) has long been accredited a physiological role in lignin degradation, but evidence to provide insights into the biochemical mechanisms and interactions is insufficient. There are ample data in the literature on the oxidase and dehydrogenase activities of POx, yet the biological relevance of this duality could not be established conclusively. Here we present a comprehensive biochemical and phylogenetic characterization of a novel pyranose 2-oxidase from the actinomycetous bacterium Kitasatospora aureofaciens (*Ka*POx) as well as a possible biomolecular synergism of this enzyme with peroxidases using phenolic model substrates *in vitro*. A phylogenetic analysis of both fungal and bacterial putative POx-encoding sequences revealed their close evolutionary relationship and supports a late horizontal gene transfer of ancestral POx sequences. We successfully expressed and characterized a novel bacterial POx gene from K. aureofaciens, one of the putative POx genes closely related to well-known fungal POx genes. Its biochemical characteristics comply with most of the classical hallmarks of known fungal pyranose 2-oxidases, i.e., reactivity with a range of different monosaccharides as electron donors as well as activity with oxygen, various quinones, and complexed metal ions as electron acceptors. Thus, *Ka*POx shows the pronounced duality of oxidase and dehydrogenase similar to that of fungal POx. We further performed efficient redox cycling of aromatic lignin model compounds between *Ka*POx and manganese peroxidase (MnP). In addition, we found a Mn(III) reduction activity in *Ka*POx, which, in combination with its ability to provide H_2_O_2_, implies this and potentially other POx as complementary enzymatic tools for oxidative lignin degradation by specialized peroxidases.

**IMPORTANCE** Establishment of a mechanistic synergism between pyranose oxidase and (manganese) peroxidases represents a vital step in the course of elucidating microbial lignin degradation. Here, the comprehensive characterization of a bacterial pyranose 2-oxidase from Kitasatospora aureofaciens is of particular interest for several reasons. First, the phylogenetic analysis of putative pyranose oxidase genes reveals a widespread occurrence of highly similar enzymes in bacteria. Still, there is only a single report on a bacterial pyranose oxidase, stressing the need of closing this gap in the scientific literature. In addition, the relatively small K. aureofaciens proteome supposedly supplies a limited set of enzymatic functions to realize lignocellulosic biomass degradation. Both enzyme and organism therefore present a viable model to study the mechanisms of bacterial lignin decomposition, elucidate physiologically relevant interactions with specialized peroxidases, and potentially realize biotechnological applications.

## Abstract (precis)

Establishment of a mechanistic synergism between pyranose oxidase and (manganese) peroxidases represents a vital step in the course of elucidating microbial lignin degradation. Here, the comprehensive characterization of a bacterial pyranose 2-oxidase from Kitasatospora aureofaciens is of particular interest for several reasons. First, the phylogenetic analysis of putative pyranose oxidase genes reveals a widespread occurrence of highly similar enzymes in bacteria. Still, there is only a single report on a bacterial pyranose oxidase, stressing the need of closing this gap in the scientific literature. In addition, the relatively small K. aureofaciens proteome supposedly supplies a limited set of enzymatic functions to realize lignocellulosic biomass degradation. Both enzyme and organism therefore present a viable model to study the mechanisms of bacterial lignin decomposition, elucidate physiologically relevant interactions with specialized peroxidases, and potentially realize biotechnological applications.

## INTRODUCTION

Auxiliary activity family 3 (AA3) of the Carbohydrate-Active enZymes Database (CAZy) (http://www.cazy.org/) comprises redox enzymes, which assist other AA family oxidoreductases or support the activity of glucoside hydrolases in lignocellulose degradation. All AA3 members belong to the glucose-methanol-choline (GMC) family of flavin-dependent oxidoreductases, and they typically are multidomain enzymes composed of a flavin-binding domain of the canonical Rossmann fold and a less preserved substrate-binding domain (1, 2). Subfamily AA3_4 comprises pyranose oxidases (POx [EC 1.1.3.10]), the phylogenetically most distantly related AA3 subfamily, which is also the most diverse with respect to structural features. POx oxidizes various monosaccharides, with glucose being preferred, but its substrate specificity is less strict than that of other members of the AA3 family.

The reaction mechanism of POx generally involves a hydride transfer from the sugar substrate, resulting in the reduction of its flavin adenine dinucleotide (FAD) cofactor to its hydroquinone form (FADH_2_), referred to as the reductive half-reaction. FADH_2_ is subsequently reoxidized in the oxidative half-reaction by a suitable electron acceptor (3). For POx, this electron acceptor can be molecular oxygen (which is reduced to hydrogen peroxide), a range of (substituted) quinones, (complexed) metal ions, or even various radicals (4). The role of POx has previously be seen in the provision of H_2_O_2_ to different peroxidases (5), yet these nonoxygen electron acceptors are often used with much higher catalytic efficiencies by POx, which points toward a potential physiological significance of reactions with such molecules (6).

The biological function of AA3-family redox enzymes—as defined by CAZy—is to act in conjunction with CAZymes during lignocellulose degradation. White-rot wood-degrading basidiomycetes employ a number of lignin-modifying enzymes (LMEs), extracellular heme-containing lignin, manganese, or versatile peroxidases as well as laccases. Hydrogen peroxide mediates the formation of the reactive peroxidase intermediate compound I [oxoiron(IV) porphyryl radical], which then—depending on the enzyme—generates various small oxidants, including the veratryl alcohol cation radical or Mn(III) coordination complexes. These diffusible mediators subsequently react with lignin in a nonspecific way, generating radical sites and thereby initiating a cascade of bond scission, which eventually results in lignin depolymerization (7–10).

Pyranose oxidase and certain other AA3 oxidases can also show a very pronounced dehydrogenase activity. Dehydrogenases involved in lignocellulose degradation are implicated in maintaining a quinone/hydroquinone redox cycle as well as in the provision of reduced metals for diverse radical-based depolymerization reactions (11). Recently, POx from the white-rot basidiomycete Irpex lacteus was shown to reduce quinoid intermediates produced by laccase from phenolic compounds and lignosulfonate *in vitro* and thus prevent their (re)polymerization (12). The same effect was also observed for this fungal POx when acting on extracted lignin with peroxidases (13). This is consistent with a proposed biological function of detoxifying lignin degradation products or phenolic compounds that are part of plant defense mechanisms (14).

Research on the enzymology of lignin depolymerization and oxidative polysaccharide degradation has largely focused on fungal systems; thus, the majority of characterized enzymes are from fungal sources (10, 15, 16), whereas knowledge on respective bacterial enzyme systems is comparably scarce. However, the capability for lignin oxidation was observed in a number of soil bacteria, the majority of which fall into the taxonomic groups of actinobacteria, alphaproteobacteria, and gammaproteobacteria (10). Recent studies implicate dye-decolorizing peroxidases (DyP) as key enzymes in bacterial lignin depolymerization (9, 17), and genome data suggest that these enzymes, while present in some fungi and higher eukaryotes, are most prominent in bacteria (18). Even though biochemical data on these bacterial enzymes are limited, it was shown that certain bacterial DyP possess a peroxidase activity comparable to those of fungal DyP and manganese peroxidases (19). Additionally, an H_2_O_2_-independent but Mn(II)- and O_2_-dependent oxidase activity was demonstrated for DyP2 from *Amycolatopsis* sp. strain 75iv2 (17).

These observations suggest that bacteria utilize mechanisms for lignin depolymerization that are more basic and “minimalistic” but similar to those used by fungi. This consequently poses several questions regarding the enzymatic equipment of these bacteria: what activities accessory to lignin and lignocellulose degradation exist and are employed in bacteria? How can lignin-modifying bacteria provide H_2_O_2_ for their peroxidases: do they possess a proprietary oxidase system for that purpose? Do bacteria utilize dehydrogenases for quinone/hydroquinone redox cycling and provision of reduced metals?

We searched for putative AA3 family enzymes in bacterial genomes by comparison with fungal AA3 sequences and established phylogenetic relationships between fungal and bacterial AA3 sequences. We further expressed, purified, and characterized a novel bacterial pyranose oxidase that demonstrates oxidase as well as dehydrogenase activities and may be involved in lignocellulose depolymerization via interaction with peroxidases, as was determined *in vitro* in this study.

## RESULTS

### Phylogenetic analysis.

In order to evaluate which well-known fungal AA3 enzymes have close relatives in bacteria, we BLAST searched representative fungal enzyme sequences for their respective most similar sequences in the bacterial domain. Subsequently, their most probable phylogenetic relation was calculated and summarized in a phylogenetic tree (see Fig. S1 in the supplemental material). We found that POx is the only AA3 enzyme that is shared among fungi and bacteria. This is evident from the close relationship of identified bacterial POx hits with the clade of fungal POx sequences and a maximal (100%) bootstrap support for this relation. All other fungal AA3 enzymes, aryl-alcohol oxidase (AAO), alcohol oxidase (AOx), cellobiose dehydrogenase (CDH), glucose dehydrogenase (GDH), glucose oxidase (GOx), and pyranose dehydrogenase (PDH), have sequence hits in bacteria that cluster among or closely with characterized bacterial choline dehydrogenases (ChDH) rather than with their fungal query sequences. None of the bacterial hits were found to cluster with bacterial cholesterol oxidases (ChOx). Two bacterial sequences from the BLAST search clustered closest to fungal CDH. Still, they displayed a high degree of difference, given a branch length of 1.7 amino acid substitutions per site and sequence identities of 26% with fungal CDH. In addition, these bacterial sequences lack a cytochrome domain and therefore cannot be considered a bacterial equivalent of fungal CDH.

**FIG 1:**
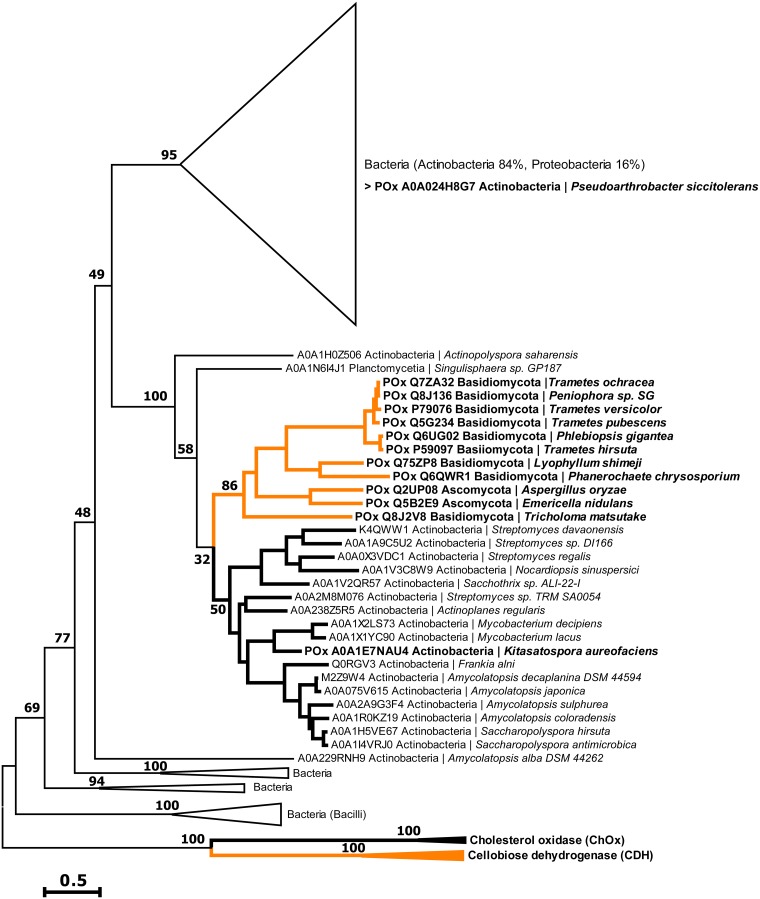
Phylogenetic tree of putative pyranose oxidase genes. The phylogenetic relation of bacterial (black) and fungal (orange) sequences based on maximum likelihood was assessed using 1,020 bootstrap repetitions as statistical support. Numbers in the graph represent bootstraps coefficients, expressed as percentages. Sequences from characterized fungal CDH and bacterial ChOx were used as outgroups. Sequences from characterized enzymes are indicated in bold letters. Most clades of closely related sequences were collapsed in triangles to reduce the complexity of the cladogram.

In the subsequent analysis of POx distribution in bacteria, we found the putative POx genes to occur mainly in *Actinobacteria* and *Proteobacteria* but also in *Bacilli* (Fig. 1). Putative POx sequences of *Proteobacteria* separated into two main clades of *Alphaproteobacteria* and *Gammaproteobacteria*, and few sequences occurred in nonspecific clades, while sequences of actinobacterial origin separated mainly into four different clades. The smallest of these four clades was found closely associated with the fungal POx sequences. Again, a small number of actinobacterial sequences were found in nonspecific clades. Finding one separate clade of sequences from *Actinobacteria* this closely related to fungal POx sequences is of high interest, especially since no sequence of this clade has been characterized so far. The putative POx sequences occurring in *Bacilli* form a single and completely separate clade.

### Sequence and structural model of pyranose oxidase from Kitasatospora aureofaciens.

We selected the putative POx from Kitasatospora aureofaciens (formerly Streptomyces aureofaciens), for which complete high-quality genome data (20) are accessible in the NCBI genome database (txid1894), for further analysis, as this sequence (*Ka*POx) was the most similar one to that from Trametes ochracea (*To*POx), the most thoroughly characterized fungal POx to date. The sequences show an identity of 38.7%, a query match of 545 out of 623 *To*POx residues, and only limited gaps in the alignment of the two sequences (Fig. S2). Assessment of potential N-terminal signal peptides in both POx sequences yielded negative outcomes for eukaryotic and Gram-positive signal predictions. Surprisingly, prediction of a bacterial twin-arginine signal peptide was positive in the fungal *To*POx sequence, comprising the initial 27 residues (Fig. S3).

The correlation of the *Ka*POx sequence alignment and a homology model (Fig. S4) showed that *Ka*POx lacks a designated presequence at the N terminus, as its first residues (starting from Y5) are already part of the canonical Rossmann fold. In contrast to that, *To*POx contains approximately 40 N-terminal residues that are not part of the Rossmann fold. A general complete match was obtained for most parts of the N-terminal Rossmann fold in *Ka*POx, the most notable difference being a missing sequence stretch (T385 to E410) corresponding to the *To*POx head domain and the absence of a loop (L337 to L349) on the opposite side of the enzyme. In addition, the *Ka*POx model displays minor deviations in the secondary-structure arrangements. Contrasting to *To*POx, the *Ka*POx homology model contains a short alpha-helix in the multimerization arm (P86 to L92).

We observed good agreement when aligning the active sites of the two POx homologues. The FAD-coordinating catalytic residues H548 and T169 in *To*POx (21) corresponded to H464 and T130 in *Ka*POx, respectively. Histidine 167, which is known to establish the covalent 8α-(N3)-histidyl link to FAD in *To*POx (22), corresponded to homologue H128 in the *Ka*POx model, presumably realizing a covalently attached FAD in the bacterial POx as well. Comparable motifs were also found for the FAD-coordinating s*i*-side helix and loop, where 122-VGGMGTHWTGAT-133 is nearly identical to the corresponding *To*POx sequence (161-VGGMSTHWTCAT-172) (differences underlined). A notable dissimilarity was found in the gating segment of the substrate recognition loop (23), where T367 (H450) and H372 (S455) were identified in the *Ka*POx model (corresponding *To*POx amino acids are in parentheses).

### Production and purification of recombinant *Ka*POx.

Shake flask cultivation of 2.5 liters E. coli suspension, carrying the *Ka*POx gene with a C-terminal 6×His tag under the control of the T7 promoter, yielded 56 g of wet cell pellet. After resuspension and cell disruption, 450 ml of crude extract (CE) was obtained and subjected to immobilized-metal affinity chromatography (IMAC) purification. Active fractions were pooled to yield 33.2 mg of purified protein, with a specific activity of 6.9 U mg^−1^ using the 2,2′-azino-bis-3-ethylbenzothiazoline-6-sulfonic acid (ABTS) assay (pH 7.5). A subsequent dialysis step buffered the protein at pH 6.0 and caused the formation of an intensely yellow precipitate. This precipitation was reversible and did not affect enzymatic activities. The presence of the 61.2-kDa *Ka*POx band could be confirmed before and after the dialysis step via SDS-PAGE (Fig. S5A). Subsequent washing of the enzyme aggregate by gentle centrifugation allowed removal of soluble impurities to yield a >95% pure *Ka*POx preparation as determined by software-aided analysis of the SDS-PAGE. Analysis by liquid chromatography-electrospray ionization mass spectrometry (LC-ESI-MS) of the washed and dialyzed enzyme confirmed its complete sequence and the absence of host cell-derived protein impurities.

### *Ka*POx is a homodimeric enzyme with covalently bound FAD.

**FIG 2:**
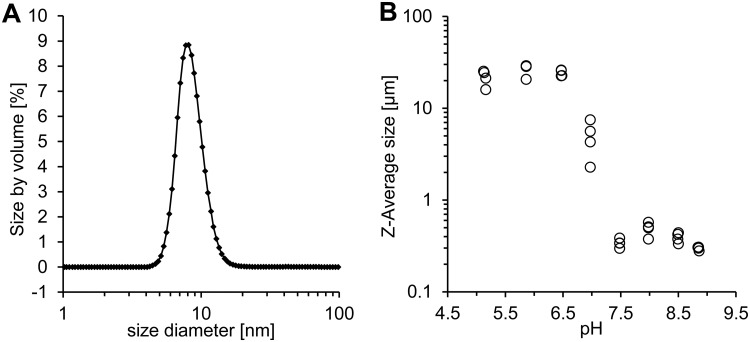
Dynamic light scattering (DLS) experiments to investigate *Ka*POx multimerization and pH-dependent aggregation. (A) Back scattering analysis of *Ka*POx. One hundred repeats were fitted to yield an average particle size (peak) of 8.5 nm for 99.7% of the mass, which estimated a protein size of 121 ± 13 kDa for the *Ka*POx at pH 8.5. (B) Protein aggregation observed during the titration of the *Ka*POx sample toward pH 5.0 was recorded by means of particle size.

Polyacrylamide gel electrophoresis under nondenaturing conditions displayed band sizes at approximately 120 kDa, indicating a 2 × 61.2 *Ka*POx dimer before and after dialysis (Fig. S5B). We observed complete resolubilization of dialyzed *Ka*POx aggregates when dissolving it in buffer at alkaline pH. Analysis of particle sizes via dynamic light scattering (DLS) revealed the pH dependence of aggregation of the purified enzyme. In accordance with native PAGE results, a dimeric state of *Ka*POx was confirmed by DLS measurements at pH 8.5. Analysis of 100 measurements yielded an estimated size distribution of 8.5 ± 1.9 nm, equaling an estimated protein size of 121 ± 13 kDa for 99.7% of the monodisperse mass (Fig. 2A). Titration of the soluble sample from pH 9.0 to 5.0 revealed initiation of aggregation at a pH below 7.5 (Fig. 2B).

Histidine 128, as was suggested by the *Ka*POx homology model, was confirmed to be covalently modified by FAD via LC-ESI-MS analysis of the chymotryptic digest of the enzyme. The covalently bound FAD moiety was identified on the peptide 121-AVGGMGTHW-129 containing the proposed H128. In the sample, a total of 91% of this peptide was conjugated with a FAD, leaving a respective 9% unmodified (Fig. S6). The same ratio was calculated by MS analysis of intact (undigested) recombinant *Ka*POx protein (data not shown).

### *Ka*POx performs C-2 site oxidation in d-glucose.

High-performance liquid chromatography (HPLC) analysis of *Ka*POx oxidation products from batch conversion experiments confirmed the characteristic oxidation of sugar substrates at the C-2 position. A mixture of d-glucose and 2-keto-d-glucose (both 25 mM) was used as a measurement standard, and retention times (*R_t_*) of 12.8 min and 15.3 min were determined, respectively. We detected a gradual increase in 2-keto-d-glucose signals and concomitant decrease of d-glucose signals (Fig. S7): after an initial lag phase, the oxidation proceeded almost linearly until the reaction was stopped at 84% d-glucose conversion after 20 h (data not shown).

### Catalytic properties of *Ka*POx.

Steady-state measurements with ambient oxygen as the electron acceptor yielded the Michaelis-Menten parameters presented in Table 1. These kinetic data display a pronounced preference for monosaccharides in general and for d-glucose and d-galactose in particular. This is predominantly reflected in the low *K_m_* values of 1.5 ± 0.1 mM and 2.7 ± 0.5 mM, respectively. Efficient turnover of L-sorbose, d-xylose, d-glucono-1,5-lactone, and d-mannose, but no specific reactivity with any of the tested disaccharides, was observed.

**TABLE 1:** Apparent kinetic constants of POx from K. aureofaciens for various electron donors^*a*^

Substrate	*V*_max_ (U mg^−1^)	Activity^*b*^ (%)	*K_m_* (mM)	*k*_cat_ (s^−1^)	*k*_cat_/*K_m_* (mM^−1^ s^−1^)
d-Glucose	7.6 ± 0.0	100	1.5 ± 0.1	15.4 ± 0.0	10.0
d-Galactose	5.8 ± 0.2	77	2.7 ± 0.5	11.9 ± 0.5	4.40
l-Sorbose	5.0 ± 0.2	66	13.6 ± 1.3	10.2 ± 0.5	0.75
d-Xylose	3.3 ± 0.2	44	32.4 ± 3.9	6.8 ± 0.3	0.21
d-Glucono-1,5-lactone	0.8 ± 0.0	10	28.7 ± 2.4	1.8 ± 0.1	0.06
d-Mannose	1.1 ± 0.1	14	201.0 ± 60.1	2.2 ± 0.3	0.01

a Data were obtained from the standard ABTS assay under standard conditions with oxygen as electron acceptor (air saturation). Values represent averages and standard deviations of 3 technical replicates. For d-glucose-1-phosphate, d-ribose, d-sorbitol, maltose, cellobiose, lactose, and sucrose, substrate observed reaction rates under assay conditions (*V*_obs_) values were <2% those for saturated d-glucose.

b Values are expressed as relative activities with respect to the *V*_max_ of d-glucose (100%).

We assessed the kinetic parameters of oxygen reduction with the help of a luminescent microsensor. This was approached by following the kinetics of consumption of dissolved oxygen by *Ka*POx from an initial concentration of 800 μM to 1 μM. However, saturation of the reaction could not be observed at these concentrations, indicating the *K_m_* for O_2_ to be close to or above the maximal soluble O_2_ concentration in this setup. Thus, it was decided to fit dissolved-oxygen curves to the integrated Michaelis-Menten equation as an estimation, which yielded an apparent *K_m_* (O_2_) of 1.1 ± 0.1 mM and a *k*_cat_ of 32.4 ± 0.7 s^−1^.

In addition to oxygen, various compounds were assessed as possible electron acceptors of *Ka*POx. Catalytic efficiencies for the two-electron acceptors 1,4-benzoquinone (1,4-BQ) and dichloroindophenol (DCIP) of 311 ± 44 and 313 ± 36 mM^−1^ s^−1^, respectively, exceeded that for oxygen (30 ± 0.8 mM^−1^ s^−1^) by approximately 1 order of magnitude, which is mainly attributed to the substantially lower *K*_m_ value. The one-electron reduction reactions evaluated for the ferrocenium ion (208 ± 19 mM^−1^ s^−1^) and the ABTS radical (309 ± 8.5 mM^−1^ s^−1^) are equally pronounced in *Ka*POx (Table 2). In addition, we found complexed Mn(III) to be a one-electron acceptor for *Ka*POx. Although the measurements did not allow the estimation of kinetic parameters—due to limitations of Mn(III) complex solubility, saturation of the reaction could not be reached—the apparent activities for Mn(III) reduction were the highest among the measured compounds. We successfully verified the specific Mn(III) reduction reaction by *Ka*POx in a separate experiment (Fig. S8).

**TABLE 2:** Apparent kinetic constants of POx from K. aureofaciens for various electron acceptors^*a*^

Substrate	*V*_max_ (U mg^−1^)	Activity^*b*^ (%)	*K_m_* (mM)	*k*_cat_ (s^−1^)	*k*_cat_/*K_m_* (s^−1^ mM^−1^)
Oxygen	15.9 ± 3.1	100	1.07 ± 0.1	32.4 ± 0.7	30
1,4-Benzoquinone	12.2 ± 0.3	77	0.08 ± 0.1	24.9 ± 7.1	311
DCIP	4.4 ± 0.5	28	0.03 ± 0.0	9.4 ± 1.1	313
Ferrocenium ion	105.0 ± 9.5	660	1.03 ± 1.0	214 ± 19	208
ABTS radical^*c*^	6.1 ± 0.2	38	0.04 ± 0.0	12.4 ± 0.3	309
Mn(III)^*d*^	226.0 ± 2.8				

a Data were obtained under standard conditions (unless indicated otherwise) using d-glucose as saturating substrate with nitrogen bubbled solutions. Values represent averages and standard deviations for 3 technical replicates.

b Values are expressed as relative activities with respect to the *V*_max_ of oxygen (100%).

c Laccase was used to prepare the ABTS cationic radical and was removed by ultrafiltration. The ABTS radical concentration was determined photometrically.

d For Mn(III), no saturation could be reached; *V*_max_ represents a *V*_obs_ reaction rate at the apparent solubility limit of the Mn(III) complex at 1 mM.

Analysis of the pH dependence of *Ka*POx activity revealed overlapping pH dependencies for the electron acceptors O_2_ and BQ (maximal activities at pH 8.0 to 8.5) and a shifted pH curve for DCIP toward a more acidic pH (maximal activities at pH 6.5 to 7.0) (Fig. S9). In general, the enzyme displayed effective turnover at pH 5.0 to 9.5. Still, reactions at pH 9.0 and higher could partially not been maintained for longer than 150 s under the given conditions. As progressive aggregation was observed for pure *Ka*POx samples below pH 7.5, the pH-dependent activities in this experiments could have been subject to decreased activity due to the comparably lower soluble concentrations of *Ka*POx in the activity assays.

### Oxidoreductive coupling between *Ka*POx and manganese peroxidase.

**FIG 3:**
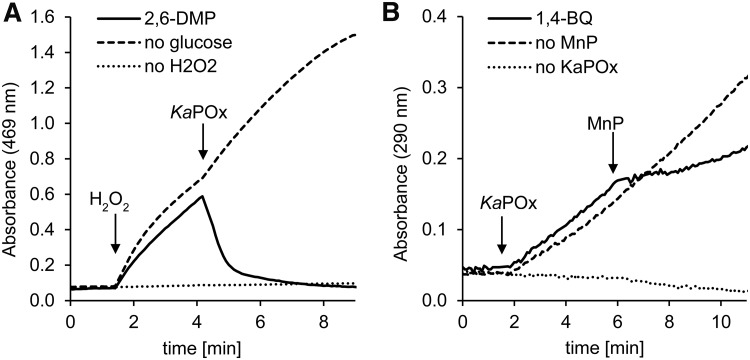
Cooperative redox cycling of substituted phenols between *Ka*POx and MnP. The qualitative photometric assays were started with phenol oxidation (A) or quinone reduction (B). (A) Assay mixtures contained manganese peroxidase (MnP), 2,6-dimethoxyphenol (2,6-DMP), and d-glucose. Reactions were started by the addition of H_2_O_2_. Four minutes into the reaction, *Ka*POx was added. (B) Assay mixtures contained 1,4-benzoquinone (1,4-BQ) and d-glucose. Reactions were started by addition of *Ka*POx; 6 min into the reaction, MnP was added.

Enzymatic redox cycling of model compounds between POx and manganese peroxidase (MnP) could be established for the methoxy-substituted phenols 2,6-dimethoxyphenol (DMP), guaiacol, acetosyringone, and sinapic acid. With the addition of hydrogen peroxide, MnP catalyzed the oxidation of these phenols to their respective quinoids, which was monitored spectrophotometrically at the respective characteristic wavelengths (Fig. 3 and Fig. S10). For 2,6-DMP and guaiacol, the subsequent addition of *Ka*POx to the assay mixture (containing the electron donor d-glucose) caused a sudden stop in oxidation and a full reversion of the reaction to base level absorbances after 5 to 7 min. For acetosyringone and sinapic acid, the addition of *Ka*POx facilitated partial reversion of the reaction, as the proceeding formation of oxidation products was stopped at a certain level after >12 min. These results indicate that the tested phenols are oxidized by MnP and are then subject to reduction by *Ka*POx.

In contrast, the *Ka*POx-mediated reduction of 1,4-benzoquinone to its reduced hydroquinone state was monitored spectrophotometrically. Upon addition of MnP a decrease in the reduction reaction was observed, clearly demonstrating that MnP catalyzed the partial reoxidation of hydroquinone, thus competing with the proceeding reduction reaction. Here, MnP catalysis is driven by H_2_O_2_ derived from the *Ka*POx reaction with d-glucose and ambient O_2_.

## DISCUSSION

In this report, we provide the biochemical characterization of a pyranose 2-oxidase from the actinomycetous, Gram-positive soil bacterium Kitasatospora aureofaciens and show a possible interaction with peroxidases using lignin model compounds as substrates *in vitro*. Since POx has been characterized from prokaryotic and eukaryotic organisms, these results will aid to compare fungal and bacterial systems for biomass degradation.

The phylogenetic analysis of sets of fungal GMC enzymes with their most similar bacterial sequences highlights the close relation between fungal and bacterial POx, as was indicated by high sequence similarities reported previously (24). In the analyzed set of GMC enzymes, the resulting putative bacterial POx sequences are more closely related to fungal POx sequences than to their most similar bacterial sequences, which is the opposite for all other GMC enzymes (Fig. S1). This strongly suggests that fungal and bacterial POxs share an ancestor. A mutual origin for bacterial and fungal POxs is also supported by the phylogenetic analysis of assembled POx sequences, where characterized fungal POx genes cluster among putative bacterial POx sequences to form a distinct clade (Fig. 1). Within this clade, bacterial actinomycetous POxs are gathered alongside their fungal homologues. POxs from Tricholoma matsutake, Aspergillus oryzae, and Aspergillus nidulans (Emericella nidulans) are the most closely related biochemically characterized fungal enzymes, with sequence similarities of >40% with respect to *Ka*POx. This supports the proposed horizontal gene transfer of bacterial POx genes into the kingdom of fungi (25). This relationship furthermore explains the often described peculiar features of fungal POx, such as the overall structural diversity and unique combination of structural motifs (14), the general lack of glycosylation (4, 26, 27), and the prediction of bacterial signal peptides (28) that separate them from other fungal GMCs.

As is observable in the phylogenetic tree of POx sequences, putative pyranose 2-oxidase-encoding genes are widely distributed in the phylum of *Firmicutes* and particularly in *Actinobacteria* (Fig. 1). In these organisms, POx would be expected to fulfill physiological roles similar to those in biomass-degrading fungi. These bacteria and fungi often share a habitat, are comparable regarding their lifestyles, and contribute to lignocellulosic biomass degradation in a synergistic manner (29–32). Hence, gene transfer between different species should be regarded as beneficial for the organisms.

The comparison of putative active-site residues of *Ka*POx with the corresponding residues of characterized fungal *To*POx shows an excellent agreement. It is therefore not surprising that the determined *Ka*POx substrate preferences are quite comparable to those of fungal POx for electron donors as well as electron acceptors. Still, *Ka*POx exhibits a pronounced reactivity with d-galactose and a distinctively high *K_m_* value for O_2_ in the low millimolar range. The ability to use oxygen as an electron acceptor seems to be less developed in bacterial POx, as was also shown for the bacterial POx from Pseudoarthrobacter siccitolerans (24). Furthermore, the kinetic characterization of *Ka*POx revealed universally reduced turnover numbers (and narrower pH dependencies) in comparison to those of fungal POx (4, 12, 33). We hypothesize that this is caused partly by differences in the gating segment of *Ka*POx, a distinct loop in the active site that was reported to be influential for the catalytic rates in fungal POx (23, 34). In *Ka*POx, the comparably bulky H372 (S455 in *To*POx) could restrict flexibility of the loop due to increased (pH-dependent) interactions with its surroundings.

Similar to other POx, *Ka*POx exhibits substantial capacity to reduce one-electron acceptors such as the ferrocenium ion and the ABTS radical. Most strikingly, we additionally confirmed the enzymatic one-electron reduction of complexed Mn(III), a reactive by-product of peroxidase activity. For *Ka*POx, turnover rates for Mn(III) and ferrocenium were the highest measured but a *K_m_* value for the reduction of Mn(III) to Mn(II) could not be resolved.

Surprisingly, we could not identify a signal peptide or targeting sequence for *Ka*POx with the available trained algorithms. As bacterial secretion systems are diverse and not entirely understood (35), an export of *Ka*POx from the cytoplasm can still be considered possible. A previous report on the actinomycetous Streptomyces olivaceoviridis documented the secretion of large cellulolytic enzyme complexes via the calcium-dependent dockerin-scaffoldin interaction, in which catalytically inactive scaffoldins bind various lytic enzymes at their dockerin domain (36). Studies of DyP from various actinobacteria describe the association of these enzymes with encapsulin to facilitate targeting of proteins via a C-terminal recognition sequence (37). Similar mechanisms could serve as means to translocate *Ka*POx and synergistic enzymes to the extracellular space in its native host. Interestingly, we found a dockerin-like motif and putative calcium-binding aspartate patches in the *Ka*POx homology model head domain, a domain which has not been ascribed a specific functionality in fungal POxs yet (38). We would like to stress at this point that *Ka*POx was selected for expression and characterization from a number of sequences from *Streptomyces* spp. and related species primarily based on sequence similarity. We cannot claim experimental evidence of actual growth on lignin of this bacterium.

**FIG 4:**
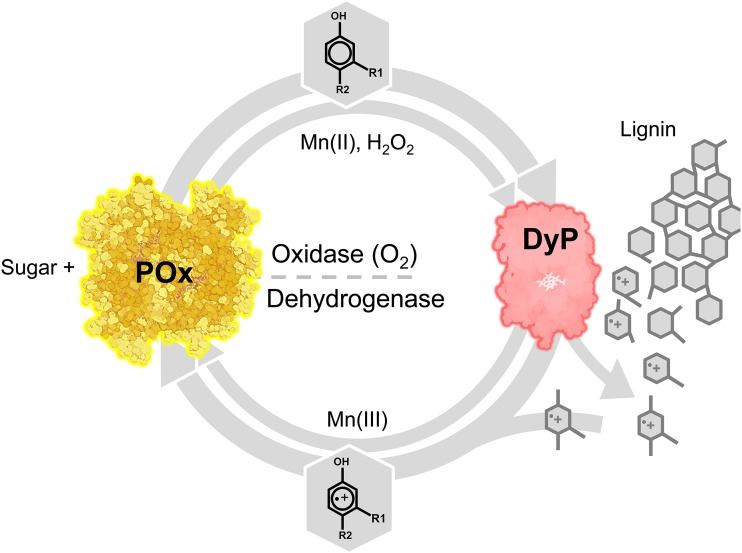
Proposed model of the physiological role of POx as a redox partner of specialized peroxidases. POx utilizes monosaccharides (d-glucose, d-galactose, d-xylose, etc.) potentially derived from (hemi)cellulose degradation. POx oxidase activity can supply diffusible H_2_O_2_ to fuel (dye-decolorizing) peroxidases (DyP). These peroxidases are known to produce aromatic radicals from lignin-derived phenols, mediating bond fission in the biopolymer. These radicals usually readily repolymerize but could be prevented from doing so if reduced by POx dehydrogenase activity as suggested. This would shift the balance toward depolymerization and additionally protect against cellular damage. For some peroxidases, Mn(III) is a by-product of their reaction. Like phenolic radicals, it can be seen as a potential mediator in depolymerization. Mn(III) can be reduced to Mn(II) and thus detoxified and recycled by POx.

As experiments with manganese peroxidase underlined, redox cycling occurs when DMP and other substituted phenols are oxidized by MnP and subsequently rereduced by *Ka*POx. We cannot experimentally verify if POx participates in the initial reduction of short-lived DMP phenoxy radicals or exclusively reduces the spontaneously formed quinoid DMP-dimer coerulignone (39, 40). Given the fact that POx efficiently mediates single-electron reductions with other substrates, we propose that aromatic radicals generally are subject to POx reduction, too. With this, the (re)polymerization of aromatic lignin constituent radicals (7–10) can be shifted toward depolymerization, as was confirmed for POx and laccase (12). The interaction of POx with manganese, and with (bacterial) DyP in particular (17, 41–43), may be even more complex. Here, the oxidase activity of POx can supply H_2_O_2_ to fuel peroxidase-mediated lignin decomposition, whereas the dehydrogenase activity recycles aromatic lignin compounds (radicals), decreases repolymerization, and scavenges highly reactive Mn(III) ions that are produced by the peroxidase (Fig. 4). A synergistic interaction between POx and peroxidases was recently demonstrated to effect lignin depolymerization *in vitro* (13).

Assuming at least limited hemicellulose degradation prior to delignification, small amounts of the major monosaccharide constituents d-glucose, d-galactose, d-xylose, l-arabinose, and d-mannose, which are all substrates for pyranose 2-oxidases, would be available at the early stage of lignocellulose deconstruction. Several reports on bacteria of the *Streptomyces* genus provide data that these organisms possess the enzymatic equipment for at least partial extracellular polysaccharide degradation down to the monosaccharide level (44–47). These monosaccharide electron donors could be oxidized by POxs with concomitant H_2_O_2_ production, while other potential electron acceptors, like lignin-derived radicals (with their higher affinity for POxs), are expected to be largely absent at this stage. Transcriptome analysis in the white-rot fungus Irpex lacteus grown on lignin indicates a role for POx in the early stages of lignin degradation as well (43). In later stages, POx may preferentially reduce the increasingly present lignin-derived radicals (thereby preventing repolymerization) and reactive Mn(III)-complexes close to the cell, where such intermediates could cause damage to cellular constituents. The “switch” between oxidase and dehydrogenase activities could simply be dictated by the concentration of the available (dehydrogenase) acceptors and the high affinity of POx for those acceptors. Based on recently reported results, however, it is also conceivable that POx-supplied H_2_O_2_ activates lytic polysaccharide monooxygenases (LPMOs) in the presence of a reductant, as was shown for the cellulose-active *Sc*LPMO10C from Streptomyces coelicolor (48). Additionally, phenols from plants and fungi were implicated in the reductive activation of LPMOs (11). This could suggest an intricate interplay of POx, peroxidase(s), and LPMO in the depolymerization of lignocellulosic substrates, particularly in the absence of a functional CDH, as we showed for bacteria.

Analysis of the K. aureofaciens genome revealed the presence of a set of genes encoding putative cellulolytic and ligninolytic enzymes. Our analysis identified five genes coding for putative laccases (26 to 27% identity to query; homologous laccase sequences are usually less conserved), six genes coding for putative DyP (42 to 61% identity to query), and five genes coding for putative LPMOs (41 to 74% identity to query). No genes with significant similarity to CAZy AA3 oxidoreductases besides POx (AOx, AAO, GOx, GDH, CDH, and PDH) could be found. Five putative choline dehydrogenases (ChDH) and two putative cholesterol oxidases (ChOx) represent the most closely related sequences to the AA3 queries (Table 3). Thus, *Ka*POx most likely represents the only AA3 family enzyme in this bacterium. Given the limited bacterial genome size (the K. aureofaciens genome is 7.1 Mb and the I. lacteus genome is 44.4 Mb), versatile oxidase and dehydrogenase activities for different purposes performed by one enzyme, POx, represent a vital advantage.

**TABLE 3:** NCBI accession numbers of K. aureofaciens genes that were identified from genome mining to encode putative enzyme functionalities in biomass degradation^*a*^

Category	Accession no. (sequence identity; E value)
AA3	
ChDH	WP_003983688.1 (40%; 2e−105)
	WP_003979475.1 (37%; 1e−101)
	WP_003981588.1 (36%; 4e−80)
	WP_003980634.1 (32%; 1e−75)
	WP_003983194.1 (31%; 1e−71)
ChOx	KOG78271.1 (82%; 0.0)
	WP_078575894.1 (83%; 0.0)
POx	WP_046385855.1 (38%; 6e−117)
DyP	WP_033347900.1 (60%; 7e−157)
	WP_050498772.1 (61%; 9e−157)
	WP_030278611.1 (60%; 1e−156)
	WP_030552786.1 (46%; 2e−95)
	WP_033348331.1 (46%; 5e−94)
	WP_003978980.1 (42%; 1e−88)
LPMO	WP_003983705.1 (74%; 2e−91)
	WP_003979198.1 (65%; 2e−83)
	WP_003986797.1 (51%; 2e−46)
	WP_033347003.1 (43%; 5e−39)
	WP_003986796.1 (41%; 1e−45)
Laccase	WP_033347195.1 (27%; 2e−42)
	WP_030552568.1 (27%; 1e−41)
	WP_003982845.1 (26%; 1e−26)
	WP_063736155.1 (26%; 2e−39)
	WP_030279851.1 (27%; 2e−38)

a Used query sequences (UniProtKB): AA3 (individual set), DyP (Q0S4I5 and K7N5M8), LPMO (Q9RJC1, Q9RJY2, B3PJ79, B3PDT6, Q838S1, Q2SNS3, C7R4I0, O83009, Q47QG3, Q47PB9, Q62YN7, and Q65N87), and laccase (J9PBQ8 and J9PBR2). No significantly similar sequence could be identified for queries of AAO, Aox, CDH, GDH, Gox, and PDH in the genome.

In summary, a comprehensive biochemical characterization of a novel pyranose 2-oxidase from the Gram-positive bacterium K. aureofaciens stresses the close biochemical similarity of this enzyme to previously reported POx from fungi. These data strongly support the close phylogenetic relation of bacterial and fungal POx established *in silico* and support the hypothesis of a late horizontal gene transfer of an ancestral POx gene from bacteria into the kingdom of fungi. The reported ability to reduce (complexed) manganese ions and the synergistic redox cycling with peroxidase by POx suggest a role in lignin degradation in bacteria.

## MATERIALS AND METHODS

### Phylogenetic analysis.

Representative sequences of characterized GMC enzymes found on the UniProtKB Protein Knowledgebase (49) and in the literature were selected to create a phylogenetic tree compiling fungal and bacterial GMC enzymes. A protein search with the Basic Local Alignment Search Tool (BLAST) in bacteria (taxid: 2) was conducted on the nonredundant NCBI protein database (50) using the following characterized fungal enzymes as query sequences: AOx (GenBank accession numbers AAB57849.1 and ABI14440.1), AAO (AMW87253.1 and ALS87663.1), PDH (AAW82997.1 and AHA85314.1), GDH (XP_002372599.1 and AIL89873.1), GOx (AGI04246.1 and AAB09442.1), CDH (ADX41688.1 and EAA27355.1), and POx (AAO13382.1 and EAA62441.1). The 10 best hits for each fungal enzyme were combined in a tree of fungal and bacterial GMC enzymes. The selections were aligned in MAFFT v7.402 (51) using the E-INS-i algorithm, and maximum likelihood trees were calculated with PhyML (52) under the LG (53) substitution model, as determined by Smart Model Selection (54) under the Akaike information criterion (AIC) selection criterion. The tree topology was optimized using nearest neighbor interchange (NNI) and subtree pruning and regrafting (SPR), and node support was assessed by performing 500 bootstrap replications.

To collect additional POx sequences occurring in bacteria we combined multiple searches on the UniProtKB, using either BLAST or HMMsearch (55). Queries for the HMMsearch were alignments of characterized fungal POx sequences and of the 10 best bacterial BLAST hits from the search described above. Queries for the BLAST search were characterized fungal POx sequences with GenBank accession numbers AAO13382 and EAA62441.1 (28, 33), characterized bacterial POx sequences with GenBank accession numbers CCQ48064.1 and A0A1E7NAU4 (6; this work), and the putative bacterial POx sequences with NCBI Reference Sequence numbers WP_028814754.1 and WP_035850787.1. All searches were restricted to E values of <1.0e−30, and duplicates were removed. Sequence names were renamed using SeqScrub (71), and the two most closely related GMC enzymes cholesterol oxidase (ChOx) and cellobiose dehydrogenase (CDH) were added as outgroups. Sequences not showing the flavin-binding GxGxxG motif (56) were removed from the selection. Sequences were aligned by MAFFT v7.402 using the FFT-NS-2 algorithm and a maximum likelihood tree was calculated with PhyML and the LG substitution model, as determined by Prottest 3.4.2 (57) under the AIC selection criterion. Tree topology was optimized using NNI and SPR and node support was assessed by performing 1,020 bootstrap replications.

### Homology model and sequence analysis of *Ka*POx.

We used the Protein Homology/Analogy Recognition Engine Phyre2 (58) to calculate the most probable homology model of the *Ka*POx based on the POx sequence from Trametes ochracaea (formerly Trametes multicolor). For this pair, a sequence identity of 38.7% is reported for a covered sequence of 545 of 623 residues. The UniProtKB protein BLAST and PyMOL 1.3 were used for analyzing both the bacterial *Ka*POx sequence (A0A1E7NAU4) and the homology model with respect to *To*POx (Q7ZA32). The SignalP online tool (59) and the TatP online tool (60) were used for predicting the presence and identity of N-terminal signal peptides.

### Recombinant expression and purification.

The full-length *Ka*POx gene was synthesized with a C-terminal 6×His tag and inserted into the pET-21b(+) expression vector, in which the standard N-terminal T7-tag was excluded (BioCat). This plasmid was then transformed into chemically competent E. coli T7 Express cells (New England BioLabs) according to the standard 5-min transformation protocol. Sequencing (Microsynth) confirmed the identity of the plasmid. The cultivation of E. coli cultures was carried out routinely in terrific broth (TB) Amp^+^ buffered at pH 7.5 and supplemented with ampicillin (100 μg ml^−1^) at 37°C. Cultures were incubated at 20°C for 20 h in the presence of 1.0% (wt/wt) lactose to induce expression of *Ka*POx. Cell disruption and immobilized metal affinity chromatography were carried out as previously described (61), with the adaptation of using 50 mM Tris-HCl based buffers at pH 8.0. Active fractions were pooled and dialyzed at 4°C against 50 mM potassium phosphate buffer (PPB; pH 6.5) using 7-kDa-cutoff Membra-Cel (Serva) dialysis tubing. After dialysis, the yellow *Ka*POx precipitate was harvested from the tube and washed twice with 50 mM PPB (pH 6.5) by centrifugation at 1,000 × *g* and 4°C for 120 s. Homogeneity of the purified protein was confirmed by SDS-PAGE and LC-ESI-MS peptide mapping.

### Protein concentration and purity.

Protein concentrations of purification fractions and pure samples were analyzed using the Bio-Rad protein assay kit according to Bradford’s method (62). For this, dilutions of bovine serum albumin were used as a standard. SDS-PAGE was carried out using Mini-PROTEAN TGX gels; Precision Plus unstained mass ladders served as a standard (both Bio-Rad). Purified protein samples were diluted to 0.5 mg ml^−1^ in 2× Laemmli buffer and incubated at 95°C for 5 min unless stated otherwise.

### PAGE under nondenaturing conditions.

Purified *Ka*POx was diluted to 1.5 mg ml^−1^ in nondenaturing sample buffer (25 mM Tris-HCl, 200 mM glycine, 10% [wt/wt] glycerol, 0.25% [wt/wt] bromphenol blue [pH 8.5]) and loaded onto a Mini-PROTEAN TGX stain-free gel (Bio-Rad) before being run at 150 V (25 to 50 mA) for 50 min. The results of the native PAGE were visualized using fluorescence imaging in a Gel Doc XR system (Bio-Rad).

### DLS analysis of *Ka*POx multimerization and aggregation.

Samples for dynamic light scattering (DLS) were prepared by diluting *Ka*POx to 1.0 mg ml^−1^ in 50 mM Tris-HCl and 100 mM NaCl (pH 8.5). Subsequently, the soluble protein solution was filtered through a 0.22-μm filter and centrifuged at 20,000 × *g* for 5 min to remove remaining aggregates. Supernatants were analyzed in a Zetasizer Nano ZSP autotitrator system (Malvern) at 633 nm, and with back scattering at an angle of 173°. Size distribution models were fitted based on data obtained from 10-s integrations of the sample (Mark-Houwink parameters: *A* = 0.428 and *k* = 7.67 × 10^−5^ cm^2^ s^−1^) with data processing optimized for protein sample by the supplier’s software. Measurements of POx from T. ochracaea and cellobiose dehydrogenase from Myceliophthora thermophila served as standards. Analysis of the pH-dependent aggregation of *Ka*POx was realized via pH titration with 0.5 M HCl from pH 9.0 to 5.0.

### Peptide profiling of *Ka*POx H128 by LC-ESI-MS.

LC-ESI-MS analysis was based on the previously described method (22) and was adapted to the given instrumentation. For localization of the covalently bound FAD, the same instruments were used as for protein identification. To this end, a total of 30 μg of *Ka*POx was S-alkylated with iodoacetamide and further digested with sequencing-grade chymotrypsin (Roche). The peptide mixture was analyzed using a Dionex Ultimate 3000 system directly linked to a quadrupole time of flight (Q-TOF) MS instrument (maXis 4G ETD; Bruker) equipped with the standard ESI source in the positive ion, data-dependent acquisition (DDA) mode (=switching to MS/MS mode for eluting peaks). MS scans were recorded (range, 150 to 2,200 *m/z*; spectrum rate, 1.0 Hz) and the six highest peaks were selected for fragmentation (collision-induced dissociation [CID] mode). Instrument calibration was performed using ESI calibration mixture (Agilent). For separation of the peptides, a Thermo BioBasic C_18_ separation column (5-μm particle size, 150 by 0.320 mm) was used. A gradient from 97% solvent A and 3% solvent B (solvent A, 65 mM ammonium formate buffer, pH 3.0 [formic acid supplied by Carl Roth; ammonia supplied by VWR BDH Prolabo]; solvent B, 80% acetonitrile [ACN; VWR BDH Prolabo] and 20% solvent A) to 40% solvent B in 45 min was applied, followed by a 15-min gradient from 40% solvent B to 95% solvent B at a flow rate of 6 μl min^−1^ at 32°C. DataAnalysis 4.0 (Bruker) was used for peptide evaluation.

### Confirmation of C-2 glucose oxidation with HPLC.

Twenty-milliliter enzymatic conversion mixes were prepared, containing 20 nM purified *Ka*POx (0.4 U), 20,000 U of washed Corynebacterium glutamicum catalase (Sigma) and 25 mM d-glucose, in 50 mM PPB (pH 7.5). Reaction mixtures were incubated at 30°C with 200 rpm shaking for 20 h; ambient oxygen served as the electron acceptor. Samples were drawn after 0, 20, 60, 180, 500, and 1,200 min, inactivated at 80°C for 20 min, and filtered through a 10-kDa spin filter prior to chromatographic analysis. 25 mM standards of d-glucose and 2-keto-d-glucose (both from Sigma) were used as analysis standards. High-performance liquid chromatography (HPLC) was carried out on a Dionex DX-500 system (Thermo Fisher) equipped with an Aminex HPX-87K column and an RI-101 refractive index detector (Shodex). Isocratic separations were run with hq-H_2_O at 0.5 ml min^−1^ (80°C) and data were processed with Chromeleon 6.5 software.

### Determination of kinetic constants.

Assessments of the catalytic properties of the *Ka*POx were commonly carried out as 300-μl colorimetric reactions in the 96 well-plate format. Britton-Robinson buffer (63) (50 mM, pH 7.5) was the standard buffer system in all measurements unless stated otherwise. Kinetic slopes were recorded at 30°C for 1,200 s using an EnSpire multimode plate reader (PerkinElmer), with measurements being performed as triplicates.

We used the established peroxidase-coupled ABTS assay (64, 65) to determine the steady-state catalytic parameters of the *Ka*POx enzyme regarding its electron-donating substrates. Assay mixes contained 0.1 μM purified *Ka*POx, 1.0 mM ABTS (ε_420_ = 36.0 mM^−1 ^cm^−1^; Amresco), horseradish peroxidase at 7.0 U ml^−1^ (181 U mg^−1^; Sigma) and 0.1 to 512 mM the respective electron donors. O_2_ at ambient concentrations (approximately 250 μM) served as the electron acceptor.

Kinetic parameters were assessed for the electron acceptors 1,4-benzoquinone (1,4-BQ), 2,6-dichloroindophenol (DCIP), the ferrocenium ion, and the cationic ABTS radical with reported wavelengths and extinction coefficients (61). Enzymatic manganese(III) reduction was carried out using 50 mM sodium malonate buffer (pH 5.5) and Mn(III) acetate to facilitate formation of a stable Mn(III)-malonate complex. Reduction of the complexed Mn(III) cation (Ɛ_270_ = 0.0116 mM^−1 ^cm^−1^) was tracked at 270 nm (66) and reactions were run at 18°C to minimize autolytic dissociation. Colorimetric assay mixes contained 30 mM d-glucose, 0.5 μM purified *Ka*POx, and a 0.001 to 4 mM concentration of the respective electron acceptor and were buffered at pH 7.5 unless stated otherwise. To minimize the interference with ambient oxygen, all solutions used in the electron acceptor kinetic experiments were bubbled with nitrogen before use. Apparent kinetic constants were estimated by nonlinear least-square regression fitting using the Microsoft Excel Solver plugin. Catalytic turnover rates are stated with respect to the dimeric form of *Ka*POx of approximately 122 kDa.

pH-dependent enzyme activities for DCIP, 1,4-BQ, and O_2_ (ABTS assay) were carried out under the aforementioned conditions and concentrations using 50 mM Britton-Robinson buffer at a pH range between 4.0 and 9.5, with increments of 0.5 unit.

We assessed the temperature-dependent inactivation of the *Ka*POx enzyme with respect to time and temperature. Buffered enzyme aliquots were incubated at 30, 36, 39, 45, 50, 56, 60, and 65°C in a C1000 thermocycler (Bio-Rad) for 20 min to evaluate temperature dependency. In contrast, a single buffered *Ka*POx sample was incubated at constant 50°C, and aliquots were drawn after 1, 2, 4, 8, 15, 20, 30, and 60 min to determine the influence of incubation times (at constant temperature) on thermal inactivation. Before measurement with the standard peroxidase/ABTS assay, samples were diluted to yield concentrations of 0.1 μM *Ka*POx in the assay mix.

### Oxygen as the electron acceptor.

The determination of apparent Michaelis-Menten parameters was realized using the luminescent oxygen microsensor Microx TX3 (PreSens), as has been described previously (67). In this way, the gradual consumption of O_2_ from the sealed reaction vial was detected. Dissolved oxygen concentrations were tracked by the sensor for 10 min (30°C) in the stirred reaction mix, which contained 1.0 μM purified *Ka*POx. Initial substrate concentrations were 100 mM and 0.850 mM for d-glucose and O_2_, respectively. The obtained oxygen consumption curves were fitted to the Runge-Kutta integration of the Michaelis-Menten equation by minimizing least-mean square errors as described previously (68).

### Quinone-hydroquinone redox cycling with manganese peroxidase.

Redox-recycling assays were carried out in 50 mM tartrate buffer (pH 5.5) and contained 0.5 mM MnCl_2_, 30 mM d-glucose, and 0.1 μM Nematoloma frowardii manganese peroxidase (MnP; Sigma), alongside 10 mM 2,6-dimethoxyphenol (DMP), 10 mM guaiacol, 0.2 mM acetosyringone, or 0.4 mM sinapic acid. Peroxidase-mediated oxidation of phenols was started by adding 0.1 mM H_2_O_2_, and reactions were run for 180 s before 1.0 μM *Ka*POx was added. In contrast, the 1,4-BQ (1 mM) reaction was started with 0.5 μM *Ka*POx before 1 μM MnP was added. Absorbance was tracked at 470 nm, 465 nm, 300 nm, 510 nm, and 290 nm, as was reported in the literature (12, 69, 70). All reactions were performed at 30°C with buffers and solutions exposed to ambient oxygen.

## Supplementary Material

Supplemental file 1
