# Supplementary material for: Versatile Oxidase and Dehydrogenase Activities of Bacterial Pyranose 2-Oxidase Facilitate Redox Cycling with Manganese Peroxidase In Vitro
Source: Appl Environ Microbiol. 2019 Jun 17;85(13):e00390-19. doi: 10.1128/AEM.00390-19 (PMC6581175; doi:10.1128/AEM.00390-19)
Supplement: Supplemental file 1 [file AEM.00390-19-s0001.pdf]

SUPPLEMENTAL INFORMATION

Supplemental figures

Figure S1.

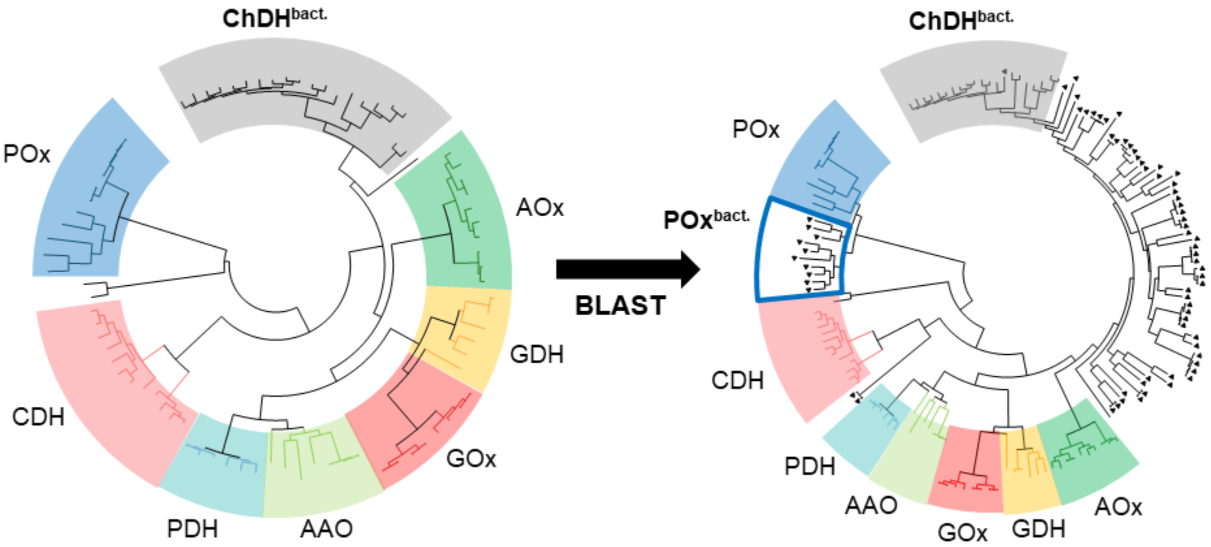

Phylogenetic indications for a horizontal gene transfer of POx. (Left) Phylogenetic tree of characterized fungal GMC_AA3 family enzyme sequences: pyranose oxidase (POx), cellobiose dehydrogenase (CDH), pyranose dehydrogenase (PDH), aryl-alcohol oxidase (AAO), glucose oxidase (GOx), glucose dehydrogenase (GDH), alcohol oxidase (AOx) and bacterial choline dehydrogenase (ChDH). (Right) Phylogenetic tree of the same collection of sequences with most similar bacterial sequences from BLAST search added.

Figure S2.

```
TR|Q7ZA32|TOPOX                MSTSSSDPFFNFAKSSFRSAAAQKASASSLPPLPGPDKKVPGMDIKYDVVIVGSGPIGCT 60
TR|A0A1E7NAU4|KAPOX            -----------------------------------------MITRYTDTLVVGSGPVGAT 19
                                                                         :    *.::*****:*.*

TR|Q7ZA32|TOPOX                YARELVGAGYKVAMFDIGEIDSGLKIGAHKKNTVEYQKNIDKFVNVIQGQLMSVSVPVNT 120
TR|A0A1E7NAU4|KAPOX            FARTLVESGREVLMVDAGAQL-SPRPGEHLKNAYIYQHNTNLFASIIRGHLHLLSVPTSA 78
                               :** ** :* :* *.* *    . : * * **:  **:* : *..:*:*:*  :***..:

TR|Q7ZA32|TOPOX                LVVDTLSP---TSWQASTFFVRNGSNPEQDPLRNLSGQAVTRVVGGMSTHWTCATPRFDR 177
TR|A0A1E7NAU4|KAPOX            RAELAVDPAAMAELGSNRSSARNAENPDQDPYRNLSAAAACYAVGGMGTHWTGATPRHHP 138
                                .  ::.*   :.  :.   .**..**:*** ****. *.  .****.**** ****..

TR|Q7ZA32|TOPOX                EQRPLLVKDDADADDAEWDRLYTKAESYFQTGTDQFKESIRHNLVLNKLTEEYKGQR--- 234
TR|A0A1E7NAU4|KAPOX            ----VL-ERYDGISDQEWDGLYGEAERLLRVSAREFDFSIRQHLVTEALRREFSELPDGY 193
                                   :* :   . .* *** ** :**  ::..: :*. ***::** : * .*:.

TR|Q7ZA32|TOPOX                DFQQIPLAAT--RRSPTFVEWSSANTVFDLQNRPNTDAPEERFNLFPAVACERVVRNALN 292
TR|A0A1E7NAU4|KAPOX            QVQSLPLAARRRRDNPRMVHWTGVDTVLGDL-----ADGHPLFSLLPQHLCTRLVLDRDG 248
                               :.*.:****   * .* :*.*:..:**:.          .  *.*:*   * *:* :  .

TR|Q7ZA32|TOPOX                SEIESLHIHDLISGDRFEIKADVYVLTAGAVHNTQLLVNSGFGQLGRPNPANPPELLPSL 352
TR|A0A1E7NAU4|KAPOX            TRIAYAEVRDLNRSETVRVVADNYVVAAGAVLAPQLLHASGIRP-------------AAL 295
                               :.*   .::**  .: ..: ** **::****   ***  **:                :*

TR|Q7ZA32|TOPOX                GSYITEQSLVFCQTVMSTELIDSVKSDMTIRGTPGELTYSVTYTPGASTNKHPDWWNEKV 412
TR|A0A1E7NAU4|KAPOX            GRYLTEHPMAFCQVILLKDLVEQARTDQRFGG--------------------------QV 329
                               * *:**: :.***.:: .:*::..::*  : *                          :*

TR|Q7ZA32|TOPOX                KNHMMQHQEDPLPIPFEDPEPQVTTLFQPSHPWHTQIHRDAFSYGAVQQSIDSRLIVDWR 472
TR|A0A1E7NAU4|KAPOX            ARHTTLFPDDDLPIPVDDPEPNVWIPVSEGRPWHAQITRDAFHYGDVPPHVDGRLIVDLR 389
                                .*   . :* ****.:****:*   .. .:***:** **** ** *   :*.***** *

TR|Q7ZA32|TOPOX                FFGRTEPKEENKLWFSDKITDAYNMPQPTFDFRFPAGRTSKEAEDMMTDMCVMSAKIGGF 532
TR|A0A1E7NAU4|KAPOX            WFGIVEPRPDNRVTFSDTRTDVMGMPQPTFEYALSPQDAE-RQHAMMAEMMRAATALGGF 448
                               :** .**: :*:: ***. **. .******:: :    :. . . **::*   :: :***

TR|Q7ZA32|TOPOX                LPGSLPQFMEPGLVLHLGGTHRMGFDEKEDNCCVNTDSRVFGFKNLFLGGCGNIPTAYGA 592
TR|A0A1E7NAU4|KAPOX            LPGSEPRFTAPGLPLHIAGTIRMGDDPQ--SSVVDTDSRVWGLENLYLGGNGVIPTGTAC 506
                               **** *:*  *** **:.** *** * :  .. *:*****:*::**:*** * ***. ..

TR|Q7ZA32|TOPOX                NPTLTAMSLAIKSCEYIKQNFTPSPFTSEAQ-------- 623
TR|A0A1E7NAU4|KAPOX            NPTLTSVAMALKAAHHLAGSREARERRRTGADEVLAVRS 545
                               *****::::*:*:..::  .         .
```

Clustal Omega sequence alignment of bacterial *Ka*POx to fungal *To*POx. The 623 amino acid long sequence of *T. ochracea* POx (TOPOX, top) was aligned to the 545 amino acid long sequence of *K. aureofaciens* POx (KAPOX, bottom) using the UniProtKB alignment tool (Clustal Omega). Identical residues at aligned positions are indicated with an asterisk (*).

Figure S3.

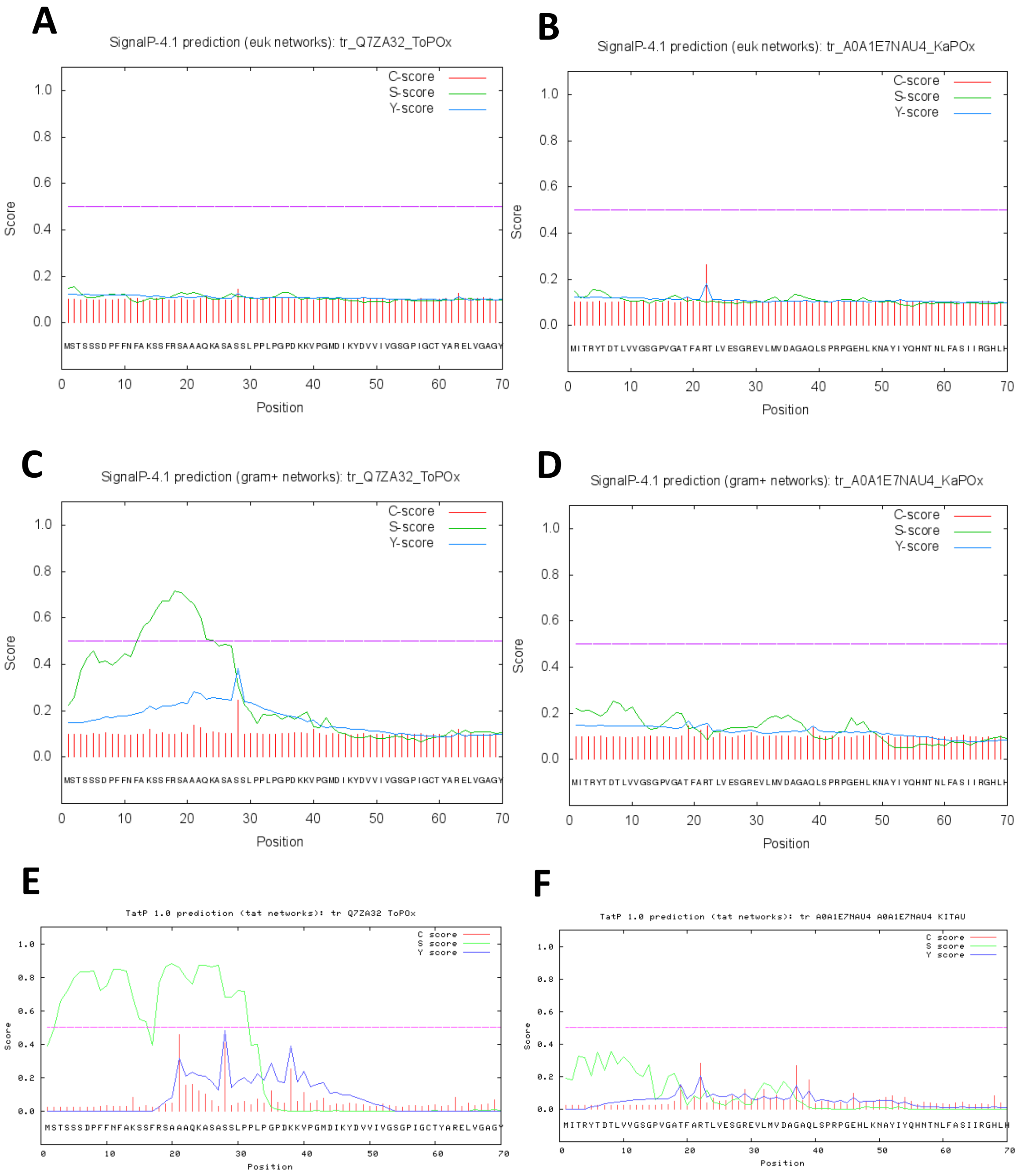

SignalP, TatP prediction of N-terminal signal peptide cleavage sites in *Ka*POx and *To*POx. The figure summarizes SignalP 4.1 signal peptide predictions from analyses of POx sequences: (A) *To*POx and (B) *Ka*POx with eukaryotic prediction; (C) *To*POx and (D) *Ka*POx with Gram-positive bacterial prediction. The TatP 1.0 online prediction tool

analyzed the first 70 residues of (E) *To*POx and (F) *Ka*POx to predict twin-arginine signal peptide cleavage sites.

Figure S4.

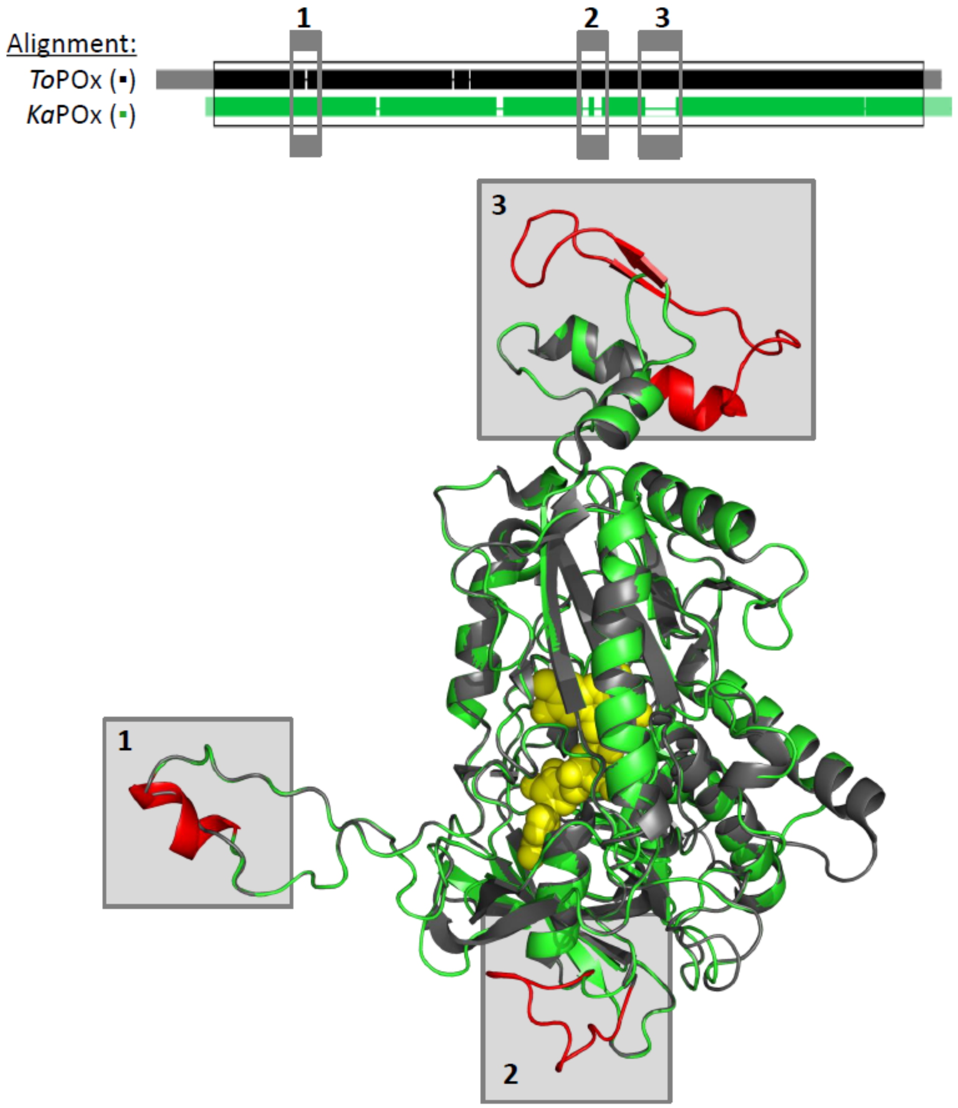

Alignment sketch and structural overlap of *To*POx and the *Ka*POx homology model. The alignment sketch (top) displays the difference of sequence length and gaps in the alignment of *To*POx and *Ka*POx. Deviations between the calculated *Ka*POx model and the *To*POx crystal structure (PDB 1TT0) are highlighted in red for both structures. The active-site FAD is displayed with a yellow sphere model. (1) An insertion in the *Ka*POx sequence with respect to *To*POx translates into a short alpha-helix in the model.

(2) Two gapped stretches in the *Ka*POx sequence translate to a shortened-surface exposed loop compared to *To*POx. (3) Another gap in the *Ka*POx sequence leaves a truncated head domain in the homology model.

Figure S5.

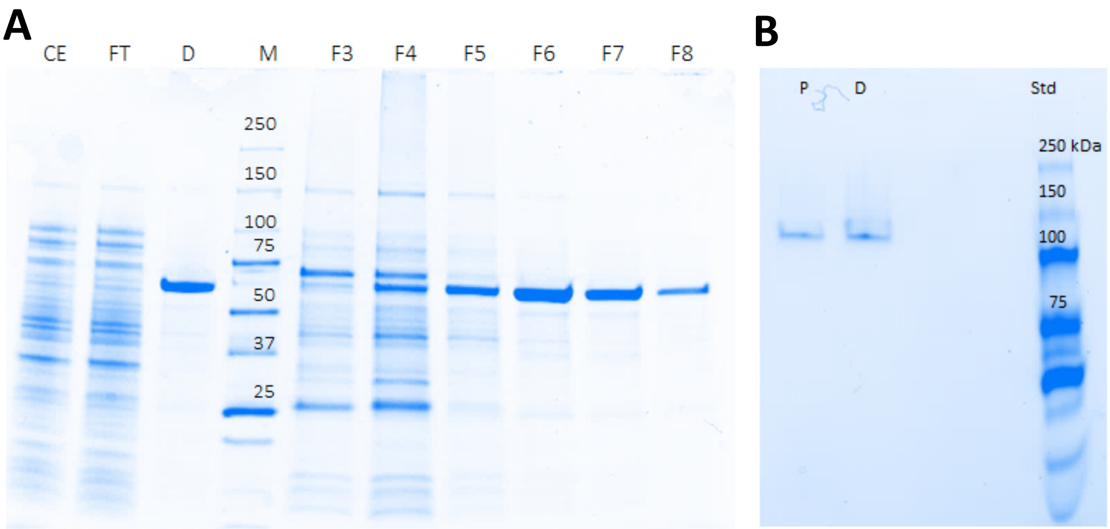

SDS-PAGE and native PAGE of *Ka*POx purification samples. (A) SDS-PAGE of IMAC purification fractions. (CE) Crude extract, (FT) flow through, (M) mass standard, (F3-F8) elution fractions, (D) dialyzed pool of fractions F6, F7, F8. (B) PAGE under non-denaturing conditions. Here, (P) represents pooled sample, (D) represents pooled and dialyzed (aggregated) sample. The numbers represent the molecular mass of the respective standard bands in kDa.

Figure S6.

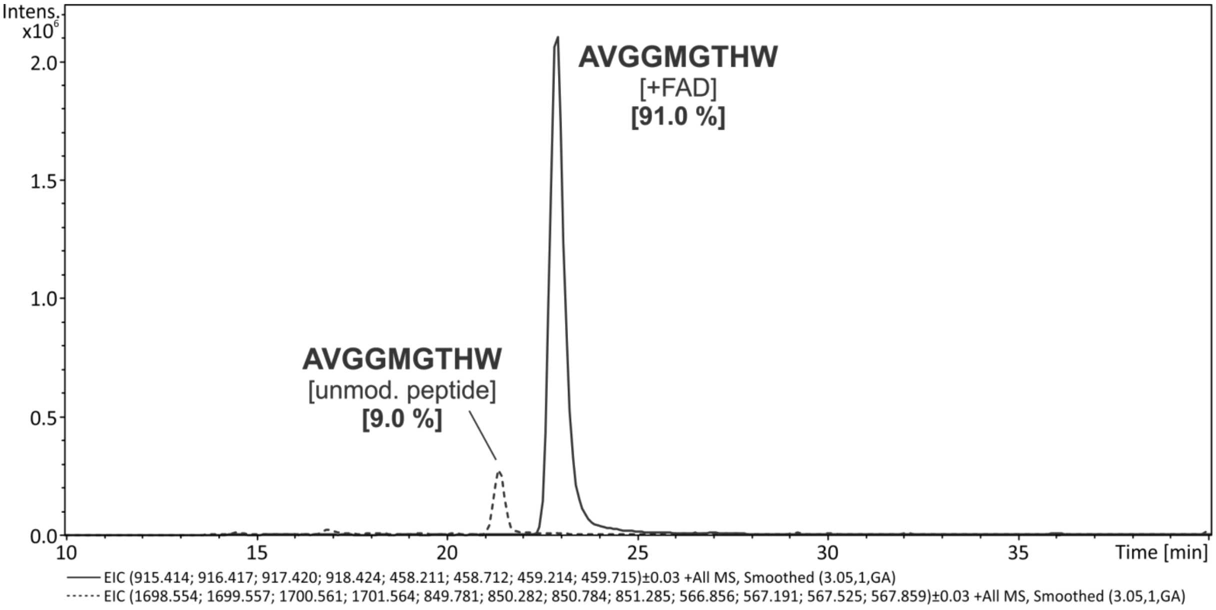

LC-ESI-MS analysis of covalent FAD attachment. Mass spectrometry resolved two different peptide masses for the 121AVGGMGTHW129 fragment after chymotryptic digest of purified *Ka*POx sample. The FAD modified peptide (black) was identified by its accurate theoretical mass and specific MS2 fragmentation profile (not shown). Additionally, a small fraction of the unmodified fragment was detected too (dashed).

Figure S7.

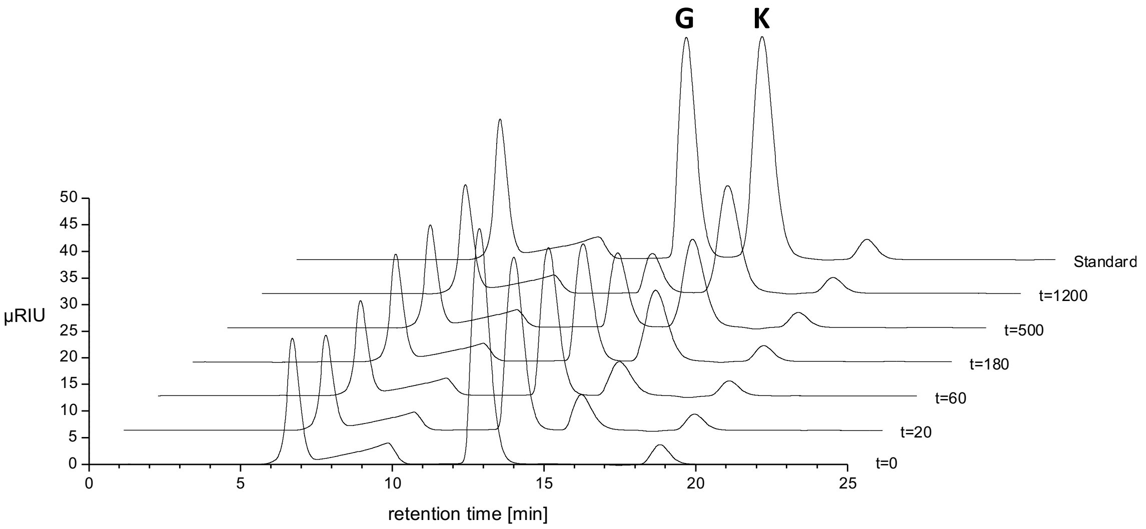

Confirmation of C2-Glucose oxidation via HPLC. Batch conversion experiments were analyzed with HPLC. D-Glucose peaks elute after a retention time of approximately 13 minutes (G), the formed C2-oxidized 2-Keto-D-glucose (K) after 15 minutes. 2-Keto-D-glucose is absent right at the reaction start (t=0) but accumulates in the reaction mix with proceeding reaction time.

Figure S8.

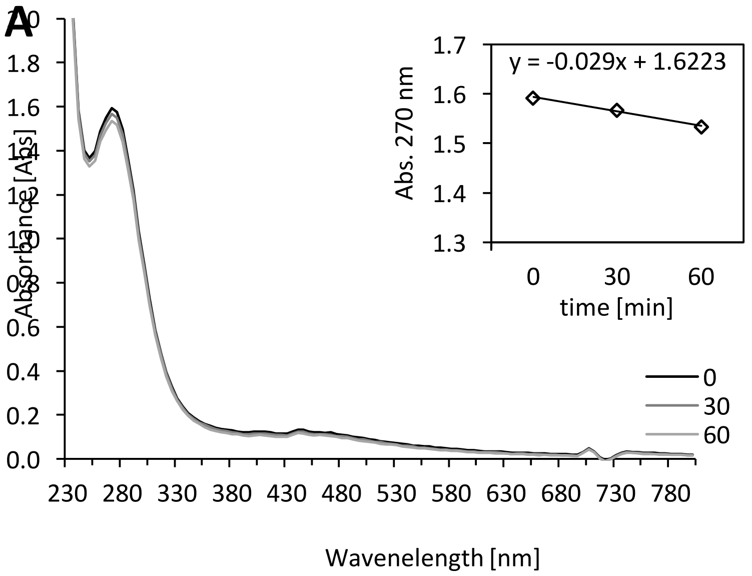
A
y = -0.029x + 1.6223
Abs. 270 nm
time [min]
0
30
60
Absorbance [Abs]
Wavenelength [nm]

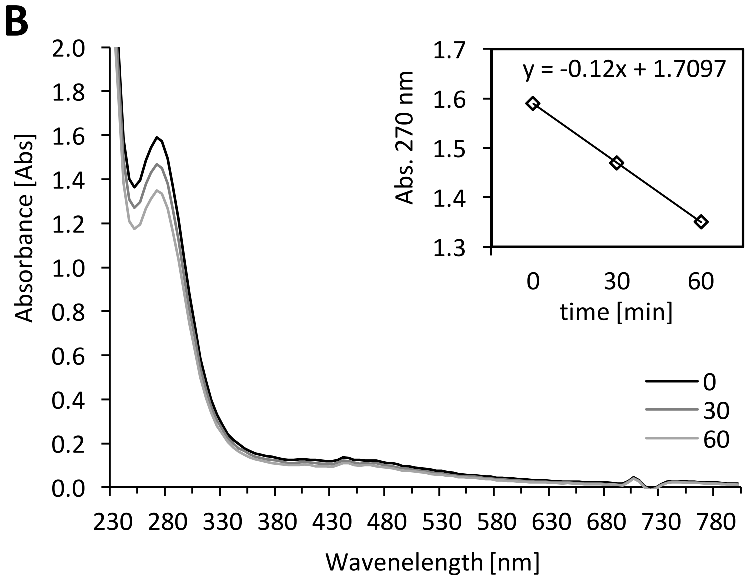
B
y = -0.12x + 1.7097
Abs. 270 nm
time [min]
0
30
60
Absorbance [Abs]
Wavenelength [nm]

C

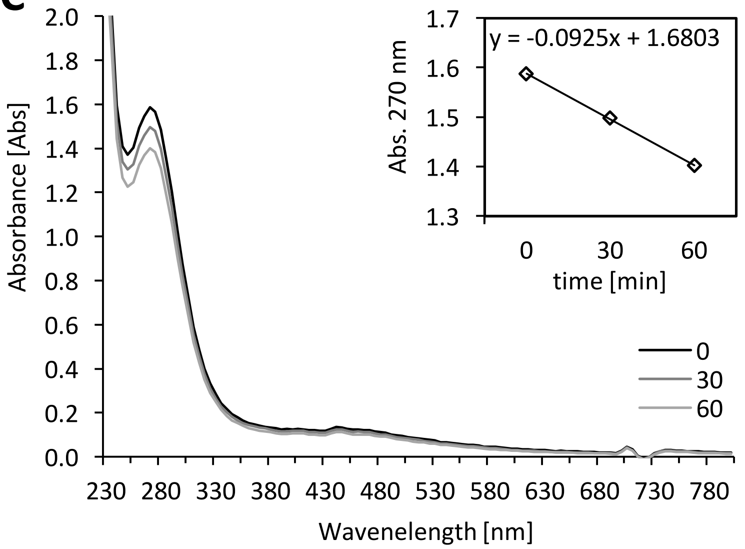
y = -0.0925x + 1.6803
Abs. 270 nm
time [min]
Absorbance [Abs]
Wavenelength [nm]
0
30
60

D

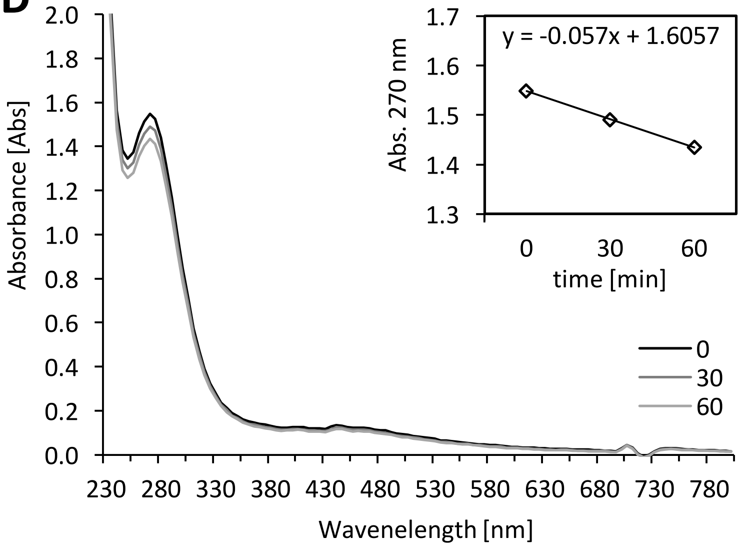
y = -0.057x + 1.6057
Abs. 270 nm
time [min]
Absorbance [Abs]
Wavenelength [nm]
0
30
60

E

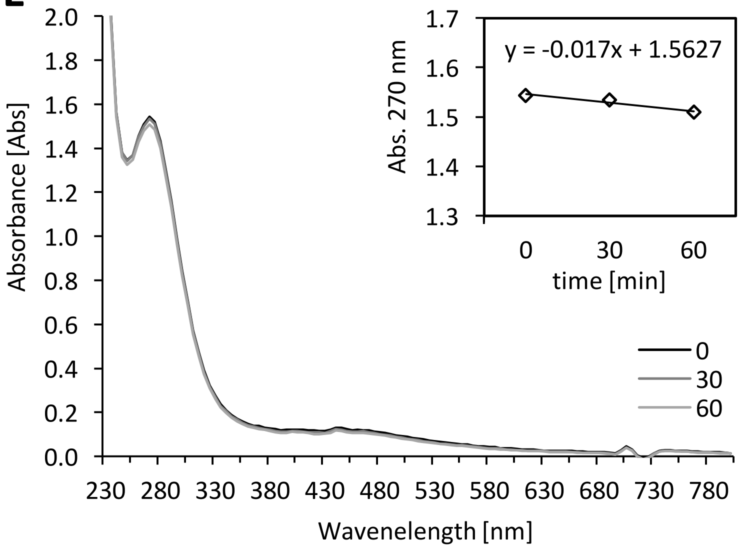

F

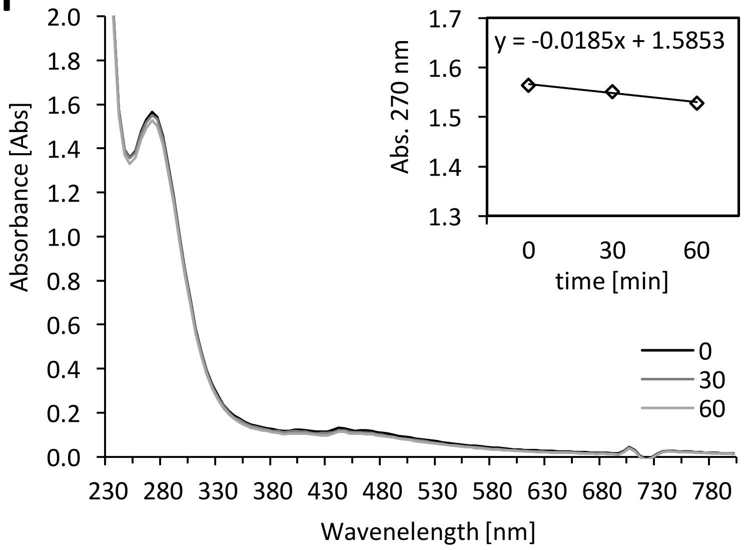

***Ka*POx dehydrogenase activity of reducing complexed Mn(III).** A 5.0 µM solution of *Ka*POx was incubated with 1.0 mM Mn(III) acetate, varying concentrations of D-glucose and 50 mM, 50 U catalase in sodium malonate buffer pH 5.5 at 18 °C. The Mn(III) malonate complexation was allowed to equilibrate for 30 min before use. Absorbance spectra were recorded (230 – 800nm) before and 20, 40 minutes into the reaction. The absorbance change at 270 nm with proceeding reaction time is observable as an insert,

with slope and intercept of data fitting indicated. (A) Instead of *Ka*POx, 5.0 µM *A. niger* glucose oxidase (Sigma) was present. (B, C. D) Concentrations of 30, 15, 8 mM D-glucose (in Buffer) were added to start the reaction, respectively. (E) Instead of D-glucose, buffer was added. (F) No *Ka*POx was present, 8 mM D-glucose were added.

Figure S9.

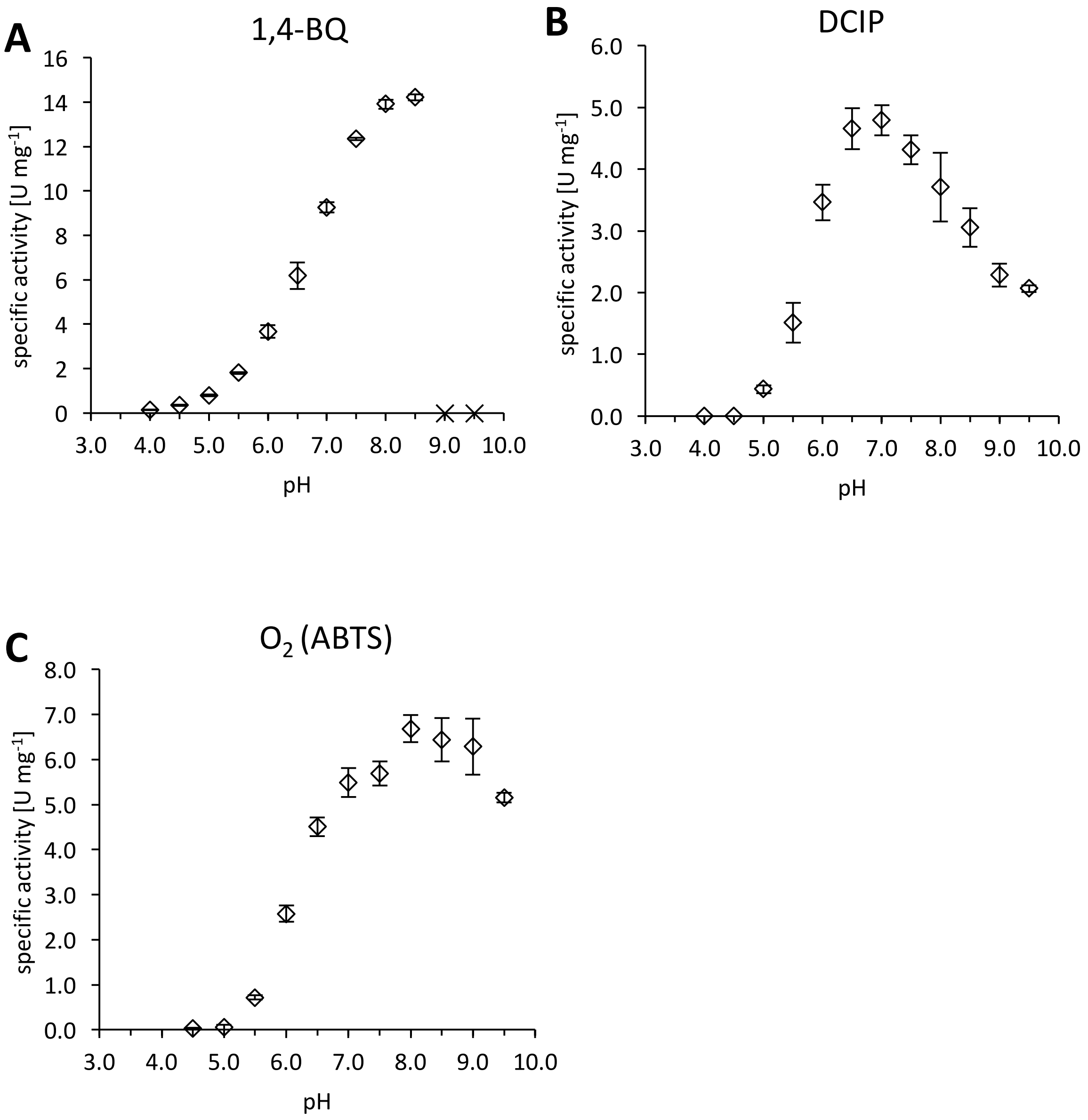

The effect of pH on the activity of *Ka*POx. Specific activities at different pHs were determined for different electron acceptors. Each value represents the average value ± standard deviation of technical triplicates. (A) 0.5 mM 1,4-benzoquinone (1,4-BQ) was used in the colorimetric assay. Reactions at pH 9.0 and 9.5 (×) were fast initially but could

not be maintained longer than 150 seconds. (B) 0.3 mM dichloroindophenol (DCIP) were used as electron acceptor. (C) Oxygen reduction was assayed with H_2O_2 production in the standard ABTS assay.

Figure S10.

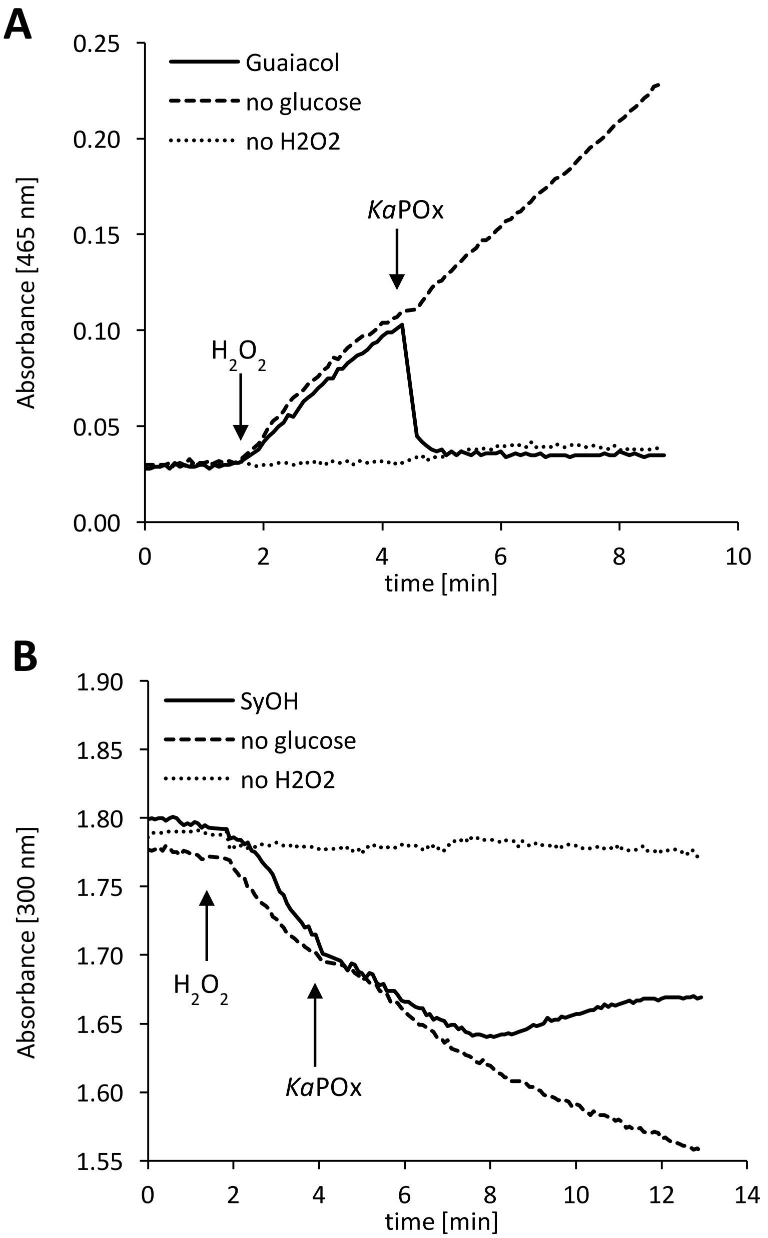
A
0.25
0.20
0.15
0.10
0.05
0.00
Absorbance [465 nm]
Guaiacol
no glucose
no H2O2
*Ka*POx
H_2O_2
0
2
4
6
8
10
time [min]
B
1.90
1.85
1.80
1.75
1.70
1.65
1.60
1.55
Absorbance [300 nm]
SyOH
no glucose
no H2O2
H_2O_2
*Ka*POx
0
2
4
6
8
10
12
14
time [min]

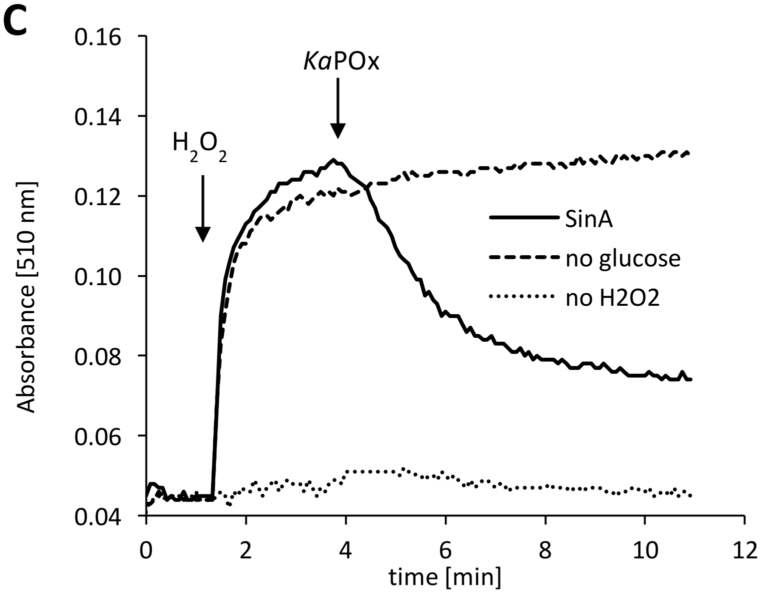

Additional redox cycling reactions of substituted phenols between *Ka*POx and MnP. Photometric assays display oxidation reactions of various substituted phenols. Assay mixtures contained manganese peroxidase (MnP), D-Glucose and the respective electron acceptor: (A) guaiacol, (B) acetosyringone (SyOH) and (C) sinapic acid (SinA). Reactions were started by the addition of H_2O_2. At approximately 4 minutes into the reaction *Ka*POx was added. Dashed line (---): no D-glucose was present. Dotted line (···): no H_2O_2 was added.
